# Islet Autoimmunity in Adults With Impaired Glucose Tolerance and Recently Diagnosed, Treatment Naïve Type 2 Diabetes in the Restoring Insulin SEcretion (RISE) Study

**DOI:** 10.3389/fimmu.2021.640251

**Published:** 2021-04-26

**Authors:** Barbara M. Brooks-Worrell, Ashley H. Tjaden, Sharon L. Edelstein, Brenda Palomino, Kristina M. Utzschneider, Silva Arslanian, Kieren J. Mather, Thomas A. Buchanan, Kristen J. Nadeau, Karen Atkinson, Elena Barengolts, Steven E. Kahn, Jerry P. Palmer, The RISE Consortium, David A. Ehrmann

**Affiliations:** ^1^Department of Medicine, University of Washington, Seattle, WA, United States; ^2^Veterans Affairs Puget Sound Health Care System, Seattle, WA, United States; ^3^Biostatistics Center, Milken School of Public Health, George Washington University Biostatistics Center, Rockville, MD, United States; ^4^Seattle Institute for Biochemical and Clinical Research, Seattle, WA, United States; ^5^Department of Medicine, University of Pittsburgh Medical Center Children's Hospital of Pittsburgh, Pittsburgh, PA, United States; ^6^Indiana University School of Medicine, Richard L. Roudebush Veterans Affairs Medical Center, Indianapolis, IN, United States; ^7^University of Southern California Keck School of Medicine/Kaiser Permanente Southern California, Los Angeles, CA, United States; ^8^University of Colorado Anschutz Medical Campus/Children's Hospital Colorado, Aurora, CO, United States; ^9^University of Chicago Clinical Research Center and Jesse Brown Veterans Affairs Medical Center, Chicago, IL, United States

**Keywords:** islet reactive T-cells, islet autoantibodies, type 2 diabetes, islet autoimmunity, pre-diabetes, impaired glucose tolerance, beta cell function, GADA

## Abstract

The presence of islet autoantibodies and islet reactive T cells (T+) in adults with established type 2 diabetes (T2D) have been shown to identify those patients with more severe β-cell dysfunction. However, at what stage in the progression toward clinical T2D does islet autoimmunity emerge as an important component influencing β-cell dysfunction? In this ancillary study to the Restoring Insulin SEcretion (RISE) Study, we investigated the prevalence of and association with β-cell dysfunction of T+ and autoantibodies to the 65 kDa glutamic acid decarboxylase antigen (GADA) in obese pre-diabetes adults with impaired glucose tolerance (IGT) and recently diagnosed treatment naïve (Ndx) T2D. We further investigated the effect of 12 months of RISE interventions (metformin or liraglutide plus metformin, or with 3 months of insulin glargine followed by 9 months of metformin or placebo) on islet autoimmune reactivity. We observed GADA(+) in 1.6% of NdxT2D and 4.6% of IGT at baseline, and in 1.6% of NdxT2D and 5.3% of IGT at 12 months, but no significant associations between GADA(+) and β-cell function. T(+) was observed in 50% of NdxT2D and 60.4% of IGT at baseline, and in 68.4% of NdxT2D and 83.9% of IGT at 12 months. T(+) NdxT2D were observed to have significantly higher fasting glucose (*p* = 0.004), and 2 h glucose (*p* = 0.0032), but significantly lower steady state C-peptide (sscpep, *p* = 0.007) compared to T(−) NdxT2D. T(+) IGT participants demonstrated lower but not significant (*p* = 0.025) acute (first phase) C-peptide response to glucose (ACPRg) compared to T(−) IGT. With metformin treatment, T(+) participants were observed to have a significantly lower Hemoglobin A1c (HbA1c, *p* = 0.002) and fasting C-peptide (*p* = 0.002) compared to T(−), whereas T(+) treated with liraglutide + metformin had significantly lower sscpep (*p* = 0.010) compared to T(−) participants. In the placebo group, T(+) participants demonstrated significantly lower ACPRg (*p* = 0.001) compared to T(−) participants. In summary, T(+) were found in a large percentage of obese pre-diabetes adults with IGT and in recently diagnosed T2D. Moreover, T(+) were significantly correlated with treatment effects and β-cell dysfunction. Our results demonstrate that T(+) are an important component in T2D.

## Introduction

Diabetes Mellitus encompasses a spectrum of disease entities with varying autoimmune involvement (1). Along one end of the spectrum is type 1 diabetes (T1D) where insulin producing beta (β)-cells are destroyed by cellular autoimmune mechanisms (2). Whereas, type 2 diabetes (T2D) has historically been considered to be on the other end of the spectrum resulting from insufficient insulin output and insulin resistance unrelated to autoimmune destruction of pancreatic β-cells. However, in recent years, evidence suggesting that islet autoimmunity is an important component of T2D has accumulated (3–6). Research has shown that innate immune cells and pro-inflammatory CD4+ T helper 1 (Th1), Th17, CD8+ T cells, and cytokines populate adipose tissue from humans with obesity, correlating with the development of systemic inflammation and the progression of metabolic disturbances, cardiovascular changes, non-alcoholic fatty liver disease, insulin resistance, and T2D (3–15). The alteration in systemic immune composition from an anti-inflammatory state in lean individuals toward a systemic pro-inflammatory state in obesity sets the stage for development of autoimmunity (3).

In established T2D, islet autoreactivity in the form of islet autoantibodies, autoantibodies to proteins associated with insulin secretion, and T cells reactive to islet proteins have been shown to be present and to correlate with more severe β-cell dysfunction (7, 16–19). In fact, attenuation of the T cell responses to islet proteins in adults with established T2D has been associated with improved C-peptide responses (20). HLA haplotypes associated with insulin resistance and the restriction of T cell receptor regions in T2D patients further suggest development of islet-targeted autoimmunity (2, 21–24). In fact, Th17 cells, effectors of autoimmune diseases, have been shown to be present in islet inflammation in both T1D and T2D, to be increased in the peripheral blood of T1D and T2D patients, and to be involved in pancreatic β-cell destruction in T1D (3, 4, 12, 15, 25, 26).

A key component of regulating autoimmunity is the balance between T cell effectors and regulatory T cells (27). In T1D and T2D, both the numbers and functions of regulatory T cells have been demonstrated to be decreased (25–28). Therefore, the involvement of the immune system, both innate and adaptive, in the development of metabolic syndromes and β-cell dysfunction/destruction in T2D is becoming more widely accepted as studies reveal the underlying metabolic and immune system interactions during disease development (4–7, 14, 17–19, 25). However, it is currently unknown at what stage during the progressive development of T2D the autoreactive immune cells are present in the peripheral blood and become associated with β-cell dysfunction.

In this ancillary study to the Restoring Insulin SEcretion (RISE) Study, a multi-site national clinical trial, we investigated the prevalence of and association with β-cell dysfunction of islet reactive T cells and autoantibodies to the 65 kDa glutamic acid decarboxylase antigen (GADA) in adults with impaired glucose tolerance (IGT), and in treatment naïve adults with recently diagnosed T2D. We further investigated the effect of 12 months of RISE treatments (metformin, liraglutide + metformin, insulin glargine followed by metformin, or placebo) on islet autoimmunity (29).

## Methods

### Study Design

Participants were recruited from the parent RISE Adult Medication Study and the metformin arm of the RISE Adult Surgery Study (BetaFat), (ClinicalTrials.gov registration numbers NCT01779362 and NCT01763346, ClinicalTrials.gov). In the RISE Adult Medication Study, participants were treated for 12 months with metformin alone, liraglutide plus metformin, 3 months of insulin glargine followed by 9 months of metformin, or placebo (29). Blood samples for the ancillary study were collected at scheduled study visits (baseline and 12 months) and shipped overnight to the University of Washington, Seattle for immune studies blinded to diabetes status and treatment group (30, 31). GADA, T cell responses to islet proteins, oral glucose tolerance tests (OGTT), and hyperglycemic clamps were performed to assess islet autoreactivity, insulin sensitivity, and β-cell function at both baseline and 12 months on treatment.

### Participants

Participants were recruited between 2013 and 2017 at four RISE Study centers participating in the adult studies (three medication sites and one surgical site). Individuals at high risk for IGT and T2D who met other study inclusion/exclusion criteria were screened with a 2-h 75-gram OGTT and HbA1c. Participants with a fasting plasma glucose 5.3–6.9 mmol/l plus an elevated 2-h glucose (>7.8 mmol/L) and Hemoglobulin A1c (HbA1c) ≤ 7.0% (53 mmol/mol) were eligible. Individuals with self-reported diabetes for <1 year and diabetes drug-naïve were also eligible. Additional details on participant recruitment and eligibility criteria have been described (29) and detailed information is available on the RISE website (https://rise.bsc.gwu.edu/web/rise/collaborators).

### β-Cell Function Protocols

Measures of glycemia and β-cell function were performed as part of the RISE parent study. The RISE study employed the oral glucose tolerance tests (OGTT) and hyperglycemic clamp. A 3-h 75-gram OGTT was performed with blood samples collected through an indwelling intravenous catheter 10 and 5 min prior to and 10, 20, 30, 60, 90, 120, 150, and 180 min after glucose ingestion (32). A two-step hyperglycemic clamp was performed on a different day following a 10-h overnight fast with goal clamped glucose levels of 11.1 and >25 mmol/L, the latter including administration of the non-glucose secretagogue arginine. These target glucose levels were achieved using boluses and a variable rate intravenous infusion of 20% dextrose, with the rate guided by a computerized algorithm developed by the RISE Consortium (33), utilizing bedside glucose monitoring. Blood samples from these tests were collected on ice, immediately separated and frozen at −80°C, and then shipped to the central biochemistry laboratory at the University of Washington, Seattle for measurement of glucose, C-peptide, and insulin.

### Hyperglycemic Clamp-Derived and OGTT Measurements

#### Insulin Sensitivity

For the hyperglycemic clamp, insulin sensitivity (M/I) was quantified as the mean of the glucose infusion rate (M) at 100, 110, and 120 min expressed per kilogram of body weight and corrected for urinary glucose loss, divided by the mean steady-state plasma insulin concentration at the same time points (34).

#### C-Peptide Responses

The acute (first phase) C-peptide response to glucose (ACPRg) was calculated as the mean incremental response above baseline (average of −10 and −5 min) from samples drawn at 2, 4, 6, 8, and 10 min after intravenous dextrose administration. Steady-state (second-phase) C-peptide concentration (SSCP) was calculated as the mean of the respective measurements at 100, 110, and 120 min of the hyperglycemic clamp. The acute C-Peptide response to arginine at maximal glycemic potentiation (ACPRmax; >25 mmol/L) was calculated as the mean concentrations in samples drawn 2, 3, 4, and 5 min after arginine injection minus the average of concentration of the samples obtained 1 and 5 min prior to arginine administration (33).

For the OGTT β-cell function analysis the inverse of fasting insulin was used as a surrogate estimate of insulin sensitivity (32). The C-peptide index (CPI) (Δ of C-peptide0–30/ Δ glucose 0–30) and insulinogenic index (IGI) (Δ insulin 0–30/ Δ glucose 0–30) were calculated using the 0- and 30-min samples from the OGTT (32–36).

### Metabolic Assays

Glucose was measured by the glucose hexokinase method using Roche reagent on a c501 autoanalyzer (Roche). C-peptide and insulin were measured by a two site immunoenzymometric assay performed on the Tosoh 2000 autoanalyzer (Tosoh Bioscience, Inc., South San Francisco, CA). The interassay coefficients of variation on quality control samples with low, medium, medium-high, and high concentrations were 2.0% for glucose, 4.3% for C-peptide, and 3.5% for insulin (33).

### Autoantibodies to the 65 kDa Glutamic Acid Decarboxylase Antigen (GADA) Assay

The GADA assay is well-established, with a sensitivity and specificity of 86 and 93% (37). For the GADA assays, samples from 213 participants were available at baseline and after 12 months of follow-up. This GADA assay participates in the Diabetes Antibody Standardization Program. GADA(+) and GADA(−) samples were included in every assay to correct for inter-assay variation, and were used to calculate an antibody index for GADA (37).

### Cellular Immunoblotting (CI, T Cell Assay)

The CI assay is well-established, with a sensitivity of 94% and a specificity of 83% (30). Cellular immunoblotting (CI) was performed on blinded blood samples shipped overnight from clinical sites. Upon receipt of the samples, the blood was subjected to Ficoll density gradient to recover the peripheral blood mononuclear cells (PBMCs). If severe hemolysis was visible and the PBMCs were observed to be of poor or insufficient quality to proceed with CI, the blood sample was discarded. PBMCs were counted and plated into 96-well plates. Nitrocellulose particles containing human islet proteins were added to the cultures, and the cultures incubated for 5 days. Tritiated-thymidine (1 mCi/well) was added, the cells were harvested 18 h later, and radioactivity measured in a β scintillation counter (LKB Pharmacia).

To prepare the nitrocellulose particles human islets were obtained from the NIH-supported Integrated Islet Distribution Program (http://iidp.coh.org). The islets were subjected to preparative one-dimensional SDS-PAGE, the gels electroblotted onto nitrocellulose, cut according to molecular weight regions, solubilized, and re-precipitated with DMSO and sodium carbonate/bicarbonate buffers. Individual molecular weight regions were assayed in triplicate for T cell stimulatory capabilities. A stimulation index (SI = cpm experimental wells/cpm control wells) >20 for the mitogen stimulation was used to define a viable sample and a positive response to tetanus toxoid was used as a positive antigen control.

In the CI assay, positive proliferative responses to islet protein blots were defined as SI ≥ 2.1 (32). PBMCs from non-diabetic controls respond to ≤ 3 blots whereas patients with autoimmune diabetes patients respond to 4–18 blots (17, 18, 30, 31). Moreover, T cell reactivity in this assay is specific for islet tissue (and not other tissues) and stable using islets obtained from different donors (30, 31).

### Islet Autoimmunity in Participants Defined

Samples at baseline and 12 months were determined to be positive or negative for GADA, GADA(+) or GADA(−), and positive or negative for T cell responses to islet proteins, T(+) or T(−), respectively. Since autoimmune markers in the peripheral blood of people have been shown to be persistent in some people and transient in others (38–41), the autoimmune status for both T2D and IGT participants was defined as follows: (a) GADA(−) or T(−) having negative responses at both time points (baseline and 12 months), and (b) GADA(+) or T(+) if they had positive results at baseline, and/or 12 months. Participants with only 1 negative time point (no second sample available) were found to be different from participants with 2 negative time points for hyperglycemic clamp measures of Steady-State C-peptide (SSCP, *p* = 0.016) and acute C-peptide response to glucose (ACPRg, *p* = 0.009). These values were lower than participants defined as negative but higher than participants classified as positive. Therefore, these samples (68/271 T cell samples and 77/290 GADA samples) were excluded from further analysis. After excluding the participants with only 1 negative time point, 213 participants were included in the final analysis for GADA. For analysis of T cell responses to islet proteins, 190 study participants had samples available at baseline and 203 participants had samples available at 12 months. Reasons for missing and/or discarded samples included inadequate blood sample volume, severe hemolysis, samples not delivered overnight, unavailable participants, and a non-viable T cell response.

### Statistical Analysis

Descriptive statistics for comparisons between baseline and 12 months for GADA(+)/GADA(−), T(+)/T(−), participants with T2D, and participants with IGT are presented as percentages or means ± *SD*. Comparisons between groups were computed using ANOVA for continuous variables and chi-square tests for categorical variables. Linear regression models were used to explore the relationship between GADA(+)/GADA(−) and T(+)/T(−) status on measures of glycemia (HbA1c, fasting glucose, 2 h glucose), insulin sensitivity (1/fasting insulin, M/I) and β-cell responses from the hyperglycemic clamp (ACPRg, SSCP, ACPRmax) and OGTT (IGI, CPI) at 12 months. To measure the effect of GADA(−)/GADA(+) and T(−)/T(+) status on month-12 β-cell functional measures, models were stratified by baseline diabetes status and adjusted for age, sex, race/ethnicity, treatment group, and baseline values. Models exploring interactions between T cell positivity and treatment group were adjusted for age, sex, race/ethnicity, and baseline BMI. β-cell response measures were also adjusted for baseline and concurrent M/I (clamp-derived insulin sensitivity). Models used natural logarithmically transformed M/I and β-cell response variables due to the skewed distribution of these data. Prior to taking logs, we added a constant of 1.06 to the ACPRg because of negative values in this β-cell response variable. All analyses were performed using SAS (SAS Institute, Cary NC). Except where noted, tests with *p* ≤ 0.01 were considered statistically significant due to the multiple analyses performed.

## Results

### Prevalence of GADA and T Cell Responses to Islet Proteins at Baseline and 12 Month Study Visits

Of the 213 participants with GADA samples at baseline, 1.6% of participants with recently diagnosed T2D and 4.6% participants with IGT were GADA(+) (Table 1). At 12 months, 1.6% of participants with T2D and 5.3% of participants with IGT were GADA(+) (Table 1).

**Table 1:** Percent positive and negative GADA and T cell responses to islet proteins in participants with T2D and participants with IGT at baseline and month-12.

	**ALL**		**T2D**		**IGT**		***p*-** values^*^
	**N**	**%**	**N**	**%**	**N**	**%**	
**Baseline**							
**GADA**	213		62		151		**0.292**
% Negative	205	96.2%	61	98.4%	144	95.4%	
% Positive	8	3.8%	1	1.6%	7	4.6%	
**T cells**	190		56		134		**0.184**
% Negative	81	42.6%	28	50.0%	53	39.6%	
% Positive	109	57.4%	28	50.0%	81	60.4%	
**Month-12+**							
**GADA**	213		62		151		**0.225**
% Negative	204	95.8%	61	98.4%	143	94.7%	
% Positive	9	4.2%	1	1.6%	8	5.3%	
**T cells**	203		57		146		**0.024**
% Negative	43	21.2%	18	31.6%	25	16.1%	
% Positive	160	78.8%	39	68.4%	121	83.9%	

* *P-values represent comparisons of participants with T2D vs. participants with IGT at baseline or 12 months using Chi Square*.

+*Negative results are negative at baseline and 12 months samples. Positive responses are positive at either baseline, month-12, or baseline and month-12*.

For the 190 participants with baseline T cell samples available, 50% of participants with recently diagnosed T2D and 60.4% of participants with IGT were T(+) at baseline (Table 1). For the 203 T cell samples available at 12 months, 68.4% of participants with T2D and 83.9% with IGT were T(+) (Table 1). There were no significant differences in demographics or anthropometric measurements between the participants who were GADA(+)/GADA(−) or T(+)/T(−) at baseline or 12-months (Supplementary Tables 1, 2).

### Relationship of GADA and T Cell Positivity With Month-12 β-Cell Function for Participants With Recently Diagnosed T2D and Participants With IGT

**Figure 1:**
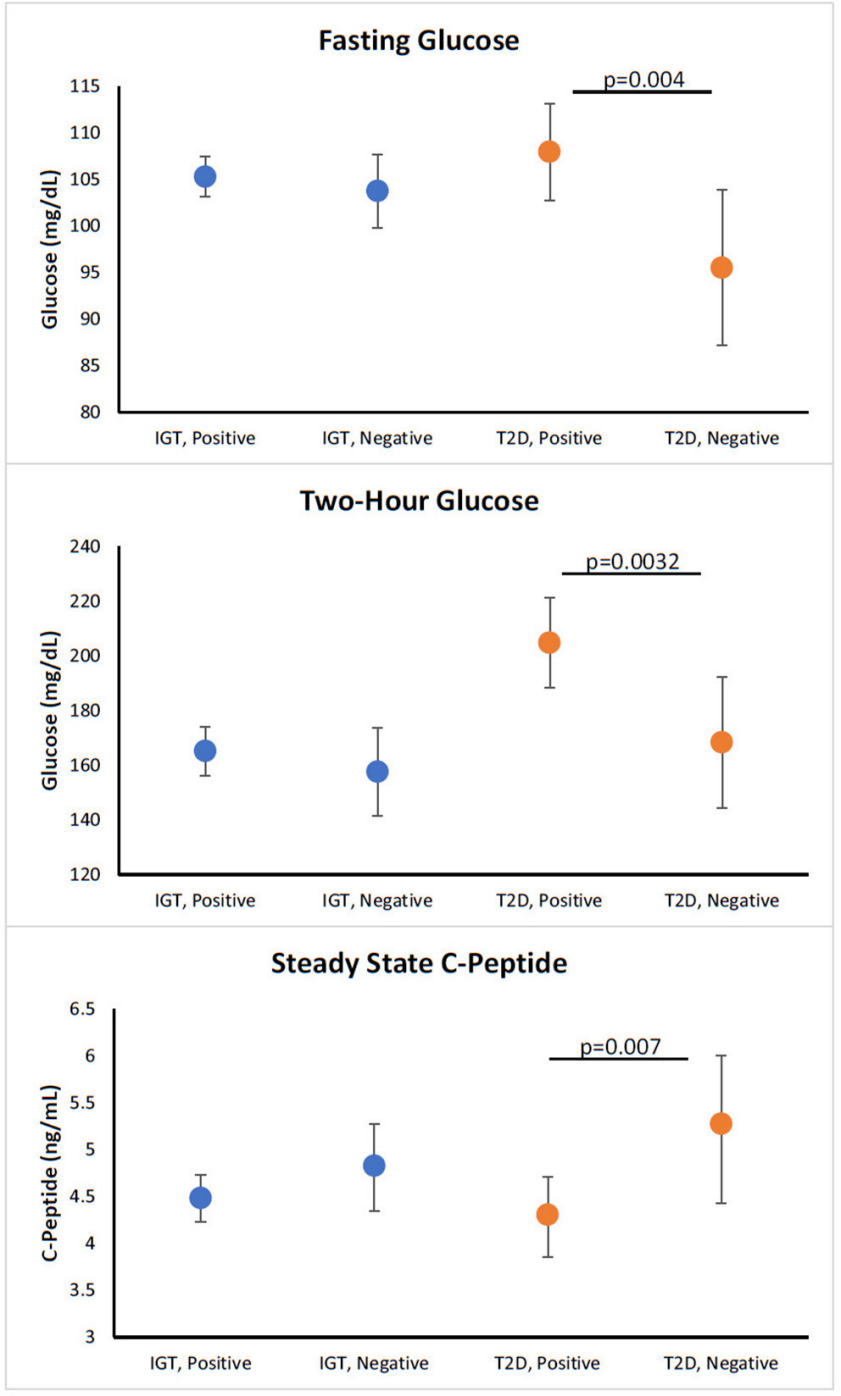
Relationship of T cell positivity with month-12 β-cell function in participants with new T2D and participants with IGT. Figures show adjusted means for values at month 12 visits. All models are adjusted for age, sex, race/ethnicity, treatment group, and baseline value of dependent variable. All models except for BMI are also adjusted for baseline BMI. Models for SSCP, and ACRPg are also adjusted for baseline and month-12 insulin sensitivity (M/I). Orange symbols represent participants with new T2D and blue symbols represent participants with IGT. *P*-value for comparison between autoimmune negative and positive for diabetes or IGT.

β-cell function at month-12 visits adjusted for baseline function was compared for participants who were GADA(−)/GADA(+) and T(−)/T(+). No significant differences were observed between GADA(−) or GADA(+) among participants with T2D or IGT (Supplementary Table 3). For T cell reactivity to islet proteins, T(+) participants with recently diagnosed T2D had significantly higher fasting glucose (*p* = 0.004), and 2-h glucose (*p* = 0.0032), but significantly lower SSCP (*p* = 0.007) compared to T(−) participants with recently diagnosed T2D (Figure 1). A complete set of data investigating the relationship of T cell reactivity with month-12 β-cell measurements (adjusted for covariates along with the baseline value), stratified by diabetes status, are summarized in Table 2.

**Table 2:** Relationship of T cell positivity with month-12 outcomes in participants with T2D and participants with IGT.

Positivity^*^	**Adjusted mean**	**95% confidence limits**		***p*-value^**†**^**
	**BMI (kg/m**^**2**^**)**			
IGT, Positive	34.31	33.91	34.72	0.2558
IGT, Negative	33.90	33.18	34.61	
T2D, Positive	32.35	31.66	33.05	0.0408
T2D, Negative	31.31	30.27	32.36	
	**HbA1c (%)**	**95% confidence limits**		***p*****-value**
IGT, Positive	5.61	5.55	5.67	0.7997
IGT, Negative	5.63	5.52	5.73	
T2D, Positive	5.62	5.43	5.81	0.0548
T2D, Negative	5.36	5.08	5.64	
	**Fasting glucose (mg/dL)**	**95% confidence limits**		***p*****-value**
IGT, Positive	105.28	103.11	107.44	0.4526
IGT, Negative	103.76	99.82	107.70	
T2D, Positive	107.95	102.78	113.13	0.0038
T2D, Negative	95.49	87.14	103.85	
	**Fasting C-peptide (ng/mL)**	**95% confidence limits**		***p*****-value**
IGT, Positive	3.18	2.91	3.45	0.2780
IGT, Negative	3.45	2.97	3.93	
T2D, Positive	3.13	2.54	3.73	0.1386
T2D, Negative	2.48	1.58	3.37	
	**Fasting insulin (uU/mL)**	**95% confidence limits**		***p*****-value**
IGT, Positive	13.03	11.06	15.00	0.1937
IGT, Negative	15.41	11.85	18.97	
T2D, Positive	11.09	5.91	16.27	0.6556
T2D, Negative	9.39	1.51	17.27	
	**2-Hr glucose (mg/dL)**	**95% confidence limits**		***p*****-value**
IGT, Positive	165.28	156.35	174.20	0.3584
IGT, Negative	157.67	141.48	173.87	
T2D, Positive	204.85	188.44	221.25	0.0032
T2D, Negative	168.36	144.21	192.51	
	**M/I (mg/kg/min/pg/L)**	**95% confidence limits**		***p*****-value**
IGT, Positive	3.54	3.04	4.12	0.8507
IGT, Negative	3.45	2.65	4.49	
T2D, Positive	3.31	2.39	4.59	0.1618
T2D, Negative	4.60	2.82	7.51	
	**Steady state C-peptide (ng/mL)**	**95% confidence limits**		***p*****-value**
IGT, Positive	4.48	4.24	4.74	0.1363
IGT, Negative	4.83	4.39	5.32	
T2D, Positive	4.31	3.90	4.75	0.0066
T2D, Negative	5.28	4.54	6.13	
	**ACPRg (ng/mL)**	**95% confidence limits**		***p*****-value**
IGT, Positive	2.10	1.99	2.22	0.0252
IGT, Negative	2.34	2.13	2.58	
T2D, Positive	1.81	1.64	2.00	0.1212
T2D, Negative	2.03	1.74	2.38	
	**ACPRmax (ng/mL)**	**95% confidence limits**		***p*****-value**
IGT, Positive	4.63	4.28	5.01	0.688
IGT, Negative	4.76	4.14	5.47	
T2D, Positive	3.51	3.12	3.95	0.419
T2D, Negative	3.77	3.14	4.52	
	**Insulinogenic index**	**95% confidence limits**		***p*****-value**
IGT, Positive	116.36	100.81	131.91	0.6699
IGT, Negative	122.49	94.05	150.94	
T2D, Positive	86.20	55.24	117.16	0.7565
T2D, Negative	93.23	45.74	140.72	
	**C-peptide index**	**95% confidence limits**		***p*****-value**
IGT, Positive	8.66	7.81	9.51	0.7407
IGT, Negative	8.92	7.39	10.46	
T2D, Positive	7.31	5.03	9.60	0.8180
T2D, Negative	7.70	4.23	11.16	

*Adjusted means for values at month 12 visits. All models are adjusted for age, sex, race/ethnicity, treatment group, and baseline value of dependent variable. All models except for BMI are also adjusted for baseline BMI. Models for SSCP, ACRPg, and ACPRmax are also adjusted for baseline and month-12 insulin sensitivity (M/I). Analyses for M/I, SSCP, ACPRg, and ACPRmax performed using the log value*.

* *Positivity over 12 months: Negative results are negative at baseline and month-12 samples. Positive responses are positive at either baseline, 12 months, or baseline and month-12*.

† *P values indicate differences between autoimmune negative and positive participants within T2D or IGT*.

### Effect of RISE Treatments on GADA and T Cell Positivity in Participants With Recently Diagnosed T2D and Participants With IGT

To investigate whether treatments had any effect on changes in GADA or T cell positivity, we compared the responses of the participants at baseline and 12 months. In participants with recently diagnosed T2D, 1 participant in the metformin only group had a GADA response which changed from GADA(+) to GADA(−) at month-12. In the participants with IGT, 1 placebo-group participant changed from GADA(−) to GADA(+) and 2 placebo-group participants changed from GADA(+) to GADA (−) at month-12. These data are summarized in Table 3.

**Table 3:** Categories of GADA status change by diabetes status and treatment group.

**Baseline-M12**	**T2D (*****N*** **=** **62)**				
	**Treatment Group**				
	**Glarg-Met**	**Metformin**	**Lira+Met**	**Placebo**	**Total**
Negative-Negative	15	18	14	14	61
Negative-Positive	0	0	0	0	0
Positive-Missing	0	0	0	0	0
Positive-Negative	0	1	0	0	1
Positive-Positive	0	0	0	0	0
Total	15	19	14	14	62
**Baseline-M12**	**IGT (*****N*** **=** **151)**				
	**Treatment group**				
	**Glarg-Met**	**Metformin**	**Lira+Met**	**Placebo**	**Total**
Negative-Negative	42	42	29	30	143
Negative-Positive	0	0	0	1	1
Positive-Missing	0	0	0	2	2
Positive-Negative	0	0	0	2	2
Positive-Positive	1	0	1	1	3
Total	43	42	30	36	151

For the T cell responses, 10 of the participants with recently diagnosed T2D were T(−) at baseline and became T(+) at month-12; 3 were in the glargine + metformin group, 5 in the metformin only group, and 2 in the placebo group. One participant in the metformin only group with T2D changed from T(+) at baseline to T(−) at month-12 (Table 4). For the participants with IGT, 28 participants changed from T(−) at baseline to T(+) at month-12; seven were in the glargine + metformin group, seven in metformin alone group, six in liraglutide + metformin group, and eight in the placebo group. Seven participants with IGT changed from T(+) at baseline to T(−) at month-12; four were in the metformin alone group, two in the liraglutide + metformin group, and one in the placebo group (Table 4).

**Table 4:** Categories of T cell status change by diabetes status and treatment group.

**Baseline-M12**	**T2D (*****N*** **=** **57)**				
	**Treatment group**				
	**Glarg-Met**	**Metformin**	**Lira+Met**	**Placebo**	**Total**
Missing-Positive	0	0	1	0	1
Negative-Negative	4	4	5	5	18
Negative-Positive	3	5	0	2	10
Positive-Missing	0	3	3	5	11
Positive-Negative	0	1	0	0	1
Positive-Positive	6	6	3	1	16
Total	13	19	12	13	57
**Baseline-M12**	**IGT (*****N*** **=** **146)**				
	**Treatment group**				
	**Glarg-Met**	**Metformin**	**Lira+Met**	**Placebo**	**Total**
Missing-Positive	4	1	3	4	12
Negative-Negative	6	5	7	7	25
Negative-Positive	7	7	6	8	28
Positive-Missing	5	12	13	4	34
Positive-Negative	0	4	2	1	7
Positive-Positive	14	11	5	10	40
Total	36	40	36	34	146

### Interaction of Autoimmune Positivity and Treatment Group on Month-12 β-Cell Function for Participants With Recently Diagnosed T2D and Participants With IGT

**Figure 2:**
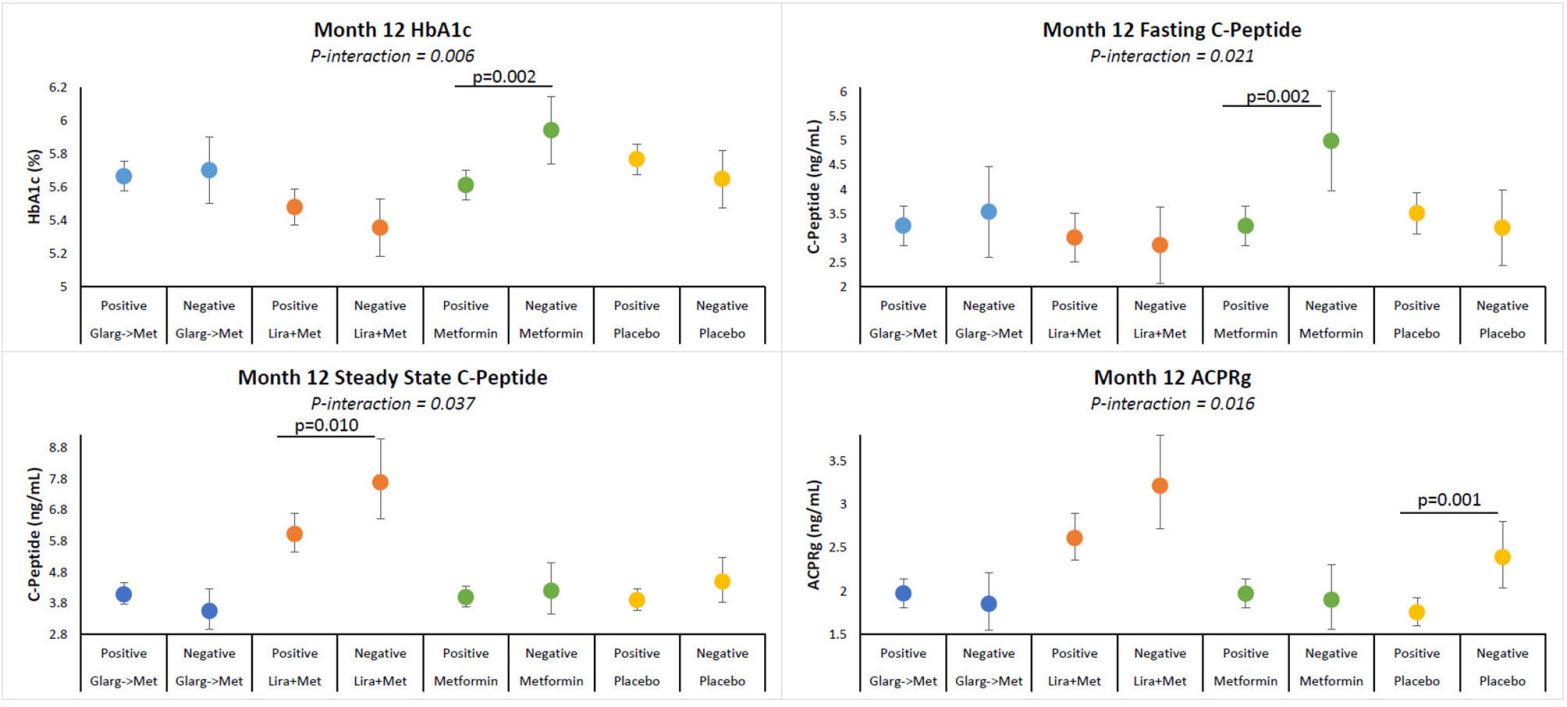
Adjusted means of HbA1c, fasting C-peptide, SSCP, and ACPRg illustrating interactions between T cell positivity and treatment group. Participants within each treatment group are identified by colors. Participants receiving 3 months of insulin glargine followed by 9 months of metformin (blue), 12 months of liraglutide + metformin (brown), 12 months of metformin (green), or placebo (yellow). Figure shows means for values at month-12 visits. All models are adjusted for age, sex, race/ethnicity, baseline insulin sensitivity, and BMI. Positivity over 12 months: Negative results are negative at baseline and month-12 samples. Positive responses are positive at either baseline, 12 months, or baseline and month-12. *P*-value for comparison between autoimmune negative and positive participants.

No significant interactions between treatment groups and GADA reactivity were found (data not shown). A significant interaction between T(+) and treatment group was identified for HbA1c (*p*-interaction = 0.006). However, no significant interactions were observed between T(+) and the other treatment groups (Figure 2). Within the metformin alone group, T(+) participants had a significantly lower HbA1c (*p* = 0.002) and fasting C-peptide (*p* = 0.002) at month-12 compared to the T(−) participants. No differences in HbA1c and fasting C-peptide between T(+) and T(−) were observed in the other treatment arms. Within the liraglutide + metformin group, T(+) had a significantly lower SSCP (*p* = 0.010) compared to T(−) participants. In the placebo group, the T(+) participants had a significantly lower ACPRg (*p* = 0.001) compared to the T(−) participants. Overall, the liraglutide + metformin group had higher SSCP and ACPRg in both T(+) and T(−) participants compared to placebo group T(+) and T(−) participants. These data are summarized in Figure 2. Adjusted means illustrating the interactions between T(+) and treatment group for all outcomes assessed are summarized in Table 5.

**Table 5:** Adjusted means of β-cell functional outcomes illustrating interactions between T cell positivity and treatment group.

**Treatment**	Positivity^*^	**Adjusted**	**95% confidence**		***p*-value^†^**
		**mean**	**limits**		
**BMI (kg/m**^**2**^**)**					***p*****-interaction=** **0.5551**
Glarg->Met	Positive	34.27	33.63	34.91	
Glarg->Met	Negative	34.44	33.07	35.81	
Lira+Met	Positive	32.98	32.19	33.77	
Lira+Met	Negative	31.83	30.56	33.09	
Metformin	Positive	34.55	33.93	35.17	
Metformin	Negative	34.55	33.09	36.02	
Placebo	Positive	35.63	34.96	36.29	
Placebo	Negative	35.03	33.81	36.26	
**HbA1c (%)**					***p*****-interaction=** **0.006**
Glarg->Met	Positive	5.664	5.575	5.753	
Glarg->Met	Negative	5.701	5.499	5.903	
Lira+Met	Positive	5.479	5.371	5.586	
Lira+Met	Negative	5.355	5.182	5.527	
Metformin	Positive	5.611	5.523	5.698	
Metformin	Negative	5.942	5.741	6.143	
Placebo	Positive	5.766	5.675	5.857	
Placebo	Negative	5.648	5.474	5.821	
**Fasting glucose (mg/dL)**					***p*****-interaction** **=** **0.2013**
Glarg->Met	Positive	106.84	103.52	110.16	
Glarg->Met	Negative	100.47	92.76	108.17	
Lira+Met	Positive	97.58	93.48	101.67	
Lira+Met	Negative	98.85	92.42	105.28	
Metformin	Positive	104.49	101.14	107.84	
Metformin	Negative	109.02	100.56	117.47	
Placebo	Positive	112.64	109.16	116.11	
Placebo	Negative	108.31	101.87	114.74	
**Fasting C-peptide (ng/mL)**					***p*****-interaction** **=** **0.021**
Glarg->Met	Positive	3.25	2.85	3.65	
Glarg->Met	Negative	3.54	2.61	4.47	
Lira+Met	Positive	3.01	2.51	3.51	
Lira+Met	Negative	2.85	2.06	3.65	
Metformin	Positive	3.25	2.84	3.65	
Metformin	Negative	5.00	3.97	6.03	
Placebo	Positive	3.51	3.09	3.94	
Placebo	Negative	3.21	2.44	3.99	
**Fasting insulin (uU/mL)**					***p*****-interaction** **=** **0.0752**
Glarg->Met	Positive	13.91	10.91	16.91	
Glarg->Met	Negative	15.22	8.24	22.20	
Lira+Met	Positive	11.91	8.21	15.61	
Lira+Met	Negative	11.86	6.07	17.66	
Metformin	Positive	13.09	10.06	16.13	
Metformin	Negative	24.25	16.61	31.89	
Placebo	Positive	14.87	11.71	18.03	
Placebo	Negative	12.80	7.10	18.50	
**2-Hr glucose (mg/dL)**					***p*****-interaction** **=** **0.4106**
Glarg->Met	Positive	175.60	161.77	189.44	
Glarg->Met	Negative	165.39	133.35	197.43	
Lira+Met	Positive	138.20	121.05	155.34	
Lira+Met	Negative	121.32	94.42	148.22	
Metformin	Positive	179.68	165.78	193.58	
Metformin	Negative	201.57	166.24	236.90	
Placebo	Positive	172.46	157.98	186.94	
Placebo	Negative	162.84	136.51	189.17	
**M/I (mg/kg/min/pg/L)**					***p*****-interaction** **=** **0.3043**
Glarg->Met	Positive	3.75	2.96	4.75	
Glarg->Met	Negative	3.05	1.83	5.09	
Lira+Met	Positive	2.76	2.06	3.70	
Lira+Met	Negative	2.63	1.66	4.16	
Metformin	Positive	3.92	3.10	4.95	
Metformin	Negative	6.41	3.74	10.99	
Placebo	Positive	4.11	3.18	5.31	
Placebo	Negative	3.92	2.49	6.18	
**Steady state C-peptide (ng/mL)**					***p*****-interaction** **=** **0.037**
Glarg->Met	Positive	4.09	3.76	4.46	
Glarg->Met	Negative	3.56	2.97	4.26	
Lira+Met	Positive	6.03	5.43	6.69	
Lira+Met	Negative	7.68	6.50	9.08	
Metformin	Positive	3.99	3.67	4.34	
Metformin	Negative	4.20	3.46	5.11	
Placebo	Positive	3.90	3.56	4.27	
Placebo	Negative	4.50	3.83	5.28	
**ACPRg (ng/mL)**					***p*****-interaction** **=** **0.016**
Glarg->Met	Positive	1.97	1.81	2.14	
Glarg->Met	Negative	1.85	1.55	2.22	
Lira+Met	Positive	2.61	2.36	2.90	
Lira+Met	Negative	3.22	2.72	3.79	
Metformin	Positive	1.97	1.81	2.14	
Metformin	Negative	1.90	1.56	2.30	
Placebo	Positive	1.76	1.60	1.92	
Placebo	Negative	2.39	2.04	2.80	
**ACPRmax (ng/mL)**					***p*****-interaction** **=** **0.7970**
Glarg->Met	Positive	5.07	4.49	5.73	
Glarg->Met	Negative	4.91	3.78	6.38	
Lira+Met	Positive	3.78	3.25	4.39	
Lira+Met	Negative	3.63	2.85	4.63	
Metformin	Positive	4.68	4.15	5.28	
Metformin	Negative	5.24	3.93	6.99	
Placebo	Positive	5.11	4.48	5.84	
Placebo	Negative	5.60	4.41	7.13	
**Insulinogenic index**					***p*****-interaction** **=** **0.4370**
Glarg->Met	Positive	118.53	94.12	142.93	
Glarg->Met	Negative	112.65	56.81	168.50	
Lira+Met	Positive	112.27	81.81	142.73	
Lira+Met	Negative	106.30	59.11	153.50	
Metformin	Positive	100.26	76.15	124.38	
Metformin	Negative	84.41	23.12	145.70	
Placebo	Positive	126.73	101.78	151.68	
Placebo	Negative	170.97	122.39	219.54	
**C-peptide index**					***p*****-interaction** **=** **0.6880**
Glarg->Met	Positive	8.43	7.07	9.78	
Glarg->Met	Negative	7.50	4.41	10.58	
Lira+Met	Positive	9.29	7.62	10.95	
Lira+Met	Negative	9.42	6.81	12.04	
Metformin	Positive	7.92	6.58	9.26	
Metformin	Negative	7.72	4.31	11.13	
Placebo	Positive	8.63	7.24	10.02	
Placebo	Negative	10.27	7.72	12.81	

*Adjusted mean for values at month-12 visits. All models are adjusted for age, sex, race/ethnicity, baseline insulin sensitivity, and BMI. Glarg->Met: Participants receiving 3 months of insulin glargine followed by 9 months of metformin, Lira+Met: 12 months of liraglutide + metformin, Metformin: 12 months of metformin alone, and Placebo: 12 months of placebo*.

* *Positivity over 12 months: Negative results are negative at baseline and 12 month samples. Positive responses are positive at either baseline, month-12, or baseline and month-12*.

† *P-values indicate interaction between treatment and positivity*.

## Conclusions

A chronic pro-inflammatory state has been demonstrated by multiple studies to be associated with obesity and is now considered to be a major driving force in T2D and associated complications (3). The upregulation of auto-reactive cells has been demonstrated to be an unfortunate result of the systemic inflammatory state (3). Islet autoimmunity in *established* T2D patients and the association with diminished β-cell function has been demonstrated by multiple studies (7, 8, 12, 15–23, 42). However, prior to this study, it was unknown if the islet autoimmune cells found in established T2D patients were present prior to clinical diagnosis (pre-diabetes). At what stage in the progression toward clinical T2D does islet autoimmunity emerge as an important component influencing β-cell dysfunction? In this ancillary study to RISE, we investigated the prevalence of GADA and islet reactive T cells and the associations between GADA and T cell reactivity to islet proteins with β-cell function in newly diagnosed T2D patients (prior to treatment) and participants without clinical T2D but with impaired β-cell function. We also investigated whether any of the RISE treatments designed to improve or preserve β-cell function affected GADA and/or islet reactive T cell responses, and whether GADA and/or islet reactive T cell positivity affected RISE treatment efficacy in participants with recently diagnosed T2D and participants with IGT.

We observed 3.8% overall positivity for GADA at baseline with the majority of the positivity observed in the IGT participants (4.6%) increasing to 5.3% in IGT participants at 12 months. For our study, we chose to focus on GADA based on previous studies in established T2D demonstrating correlations between GADA positivity and worse β-cell function (16). The GADA results in participants with recently diagnosed T2D appeared to be at a lower prevalence compared to published results in established T2D, but similar as reported in participants with IGT (38, 42, 43). We also found no correlation between GADA positivity and β-cell dysfunction in IGT or newly diagnosed T2D participants. In an 11 years longitudinal study conducted by Sørgjerd et al. (42), these researchers followed 794 participants identified to be at risk of T2D development and observed no significant associations between β-cell function, insulin sensitivity, or insulin resistance and autoantibody positivity which is consistent with our observations. In another study, Tiberti et al. (43) concluded, based on their results, that different antigen specificities may be important in the autoantibody response during different stages of T2D disease progression. This phenomenon of changing antigen specificities may be one underlying mechanism for relapsing/remitting stages of autoimmune disease both in autoantibody and cellular autoimmune compartments (38–42, 44).

For islet reactive T cells, we observed 50% of NdxT2D and 60.4% of IGT participants to be positive at baseline and this percentage increased substantially at 12 months for both NdxT2D and IGT participants, 68.4 and 83.9%, respectively. For islet reactive T cells, participants with recently diagnosed T2D demonstrated prevalence of T(+) similar to that reported in people with established T2D (16–18). Prior to this study, no reports have been published investigating islet reactive T cells in participants with recently diagnosed drug naïve T2D or participants with IGT. The high prevalence of islet reactive T cells in participants with IGT was unexpected. Changes in antigen specificities of T cells, changes in autoantibody specificities, and changes in responding cellular populations have been reported to underlie the maturation of autoimmune responses in T2D (42, 44). These changes in autoimmune reactivity may be responsible for the differences observed between participants with IGT, recently diagnosed T2D, and established T2D. One prominent advantage of our T cell assay is the inclusion of a large array of islet proteins, allowing for the identification of T cell reactivity to multiple islet proteins potentially limiting the impact of changing antigen specificity during disease progression and potentially stimulating multiple cellular populations. The stimulation of various cellular populations and cells with varying specificities may allow us to identify more subjects in different stages of disease development. This advantage may account for the high level of T cell positivity in participants with IGT. Assaying multiple autoantibodies as T2D progresses, not only GADA as performed in this study, may also identify a higher prevalence of humoral autoreactivity. Our data also suggest that islet autoimmunity in T2D may be overlooked if only one time point is assayed, only one autoantibody tested, or one islet antigen used to determine autoimmune status of a subject. Moreover, future studies identifying varying antigen specificities of cellular populations associated with different stages of disease progression, may help to uncover important biomarkers associated with early β-cell functional decline.

In this study, looking at the T cell responses (Figure 1), we observed that T(+) participants with T2D had a significantly higher fasting glucose, higher 2 h glucose, and a lower steady state C-peptide, whereas, the T(+) participants with IGT trended toward a lower ACPRg. These results in participants with recently diagnosed T2D demonstrate that T(+) participants have worse β-cell dysfunction compared to T(−) participants correlating with previous studies in people with established T2D (17–19). We did not observe any significant associations between β-cell dysfunction and GADA positivity in either adults with recently diagnosed T2D or IGT. Taken together, these results suggest that cellular islet autoimmunity is an important part of T2D. Moreover, cellular islet autoimmunity is present in the very early stages of β-cell functional decline and is associated with fasting and post-prandial hyperglycemia and decreased β-cell function.

Significant interactions were observed between T(+) and treatment groups for both participants who were recently diagnosed with T2D and participants with IGT (Figure 2), but not between GADA and RISE treatments. Of interest, was the interaction between liraglutide + metformin for both T(+) and T(−) participants. Even though T(+) was associated with significantly lower SSCP and a trend toward lower ACPRg compared to T(−), liraglutide + metformin was associated with higher SSCP and ACPRg in the T(+) and T(−) compared to placebo treated participants (Figure 2). Liraglutide has been reported to potentially have anti-inflammatory properties and have positive effects on diabetes disease management (32, 33, 45, 46). While the current study examining treatment effects was exploratory, future studies will be needed to investigate whether liraglutide is indeed beneficial to participants with cellular islet autoreactivity.

Changes in GADA and T-cell positivity were observed in both groups of participants between baseline and 12 months. These changes, either from negative to positive or positive to negative were observed to be present among all four of the RISE treatment groups. Therefore, we concluded that the changes observed in GADA and T cell reactivity between baseline and 12 months were most likely not a result of the RISE treatments. Longer follow-up of the participants with newly diagnosed T2D and IGT will help to further clarify the importance of potentially transient vs. persistent islet autoreactivity in T2D.

Lastly, the RISE clinical trial reported that the progression of T2D in youth is more rapid than in adults (46). Previously, we have identified the presence of T(+) in children with T2D (47), however in this ancillary study we were unable to incorporate children into the study due to the blood sample demands associated with the RISE clinical trial. In general, as individuals age immune responses decline (48). Therefore, it would be of interest in future studies to investigate islet cellular autoimmunity and its relationship to β-cell function in youth to determine if the presence of islet reactive T cells play a role in the accelerated progression of T2D in youth

In summary, this is the first study to show that T cells capable of recognizing islet proteins are present in a large percentage of adults with very early β-cell functional decline and that the presence of the cellular autoimmunity is associated with a detrimental effect on β-cell function. This study supports the previous studies in established T2D and suggests that immunomodulatory therapies aimed at controlling cellular islet autoimmunity may be important in treatment for T2D.

## Data Availability Statement

The raw data supporting the conclusions of this article will be made available by the authors, without undue reservation.

## Ethics Statement

The studies involving human participants were reviewed and approved by the Institutional Review Boards of all centers and all participants provided written informed consent prior to initiation of study-related activities. All participants gave written informed consent for the ancillary study, consistent with the Helsinki Declaration, and the guidelines of each center's institutional review board. The patients/participants provided their written informed consent to participate in this study.

## Author Contributions

BB-W, BP, JP, SE, and AT were responsible for research data and analysis as well as drafting and reviewing the manuscript. KU, SA, KM, TB, KN, KA, EB, and SK were responsible for clinical participant data as well as reviewing and editing the manuscript. All authors participated in the editing of this manuscript.

## Conflict of Interest

The content of this publication is solely the responsibility of the authors and does not necessarily represent the official views of the National Institutes of Health. A complete list of Centers, investigators, and staff can be found in the Appendix. At the time of production of this manuscript, KM was an employee of Eli Lilly and Company. His involvement in the research underlying this manuscript preceded this employment, and his participation in data review and preparation of the final report was independent of his employment at Eli Lilly and Company. The remaining authors declare that the research was conducted in the absence of any commercial or financial relationships that could be construed as a potential conflict of interest.

## Appendix

### Rise Consortium Investigators

**University of Chicago Clinical Research Center and Jesse Brown VA Medical Center (Chicago, IL)**

David A. Ehrmann, MD*
Karla A. Temple, PhD, RD+
Abby Rue**
Elena Barengolts, MD
Babak Mokhlesi, MD, MSc
Eve Van Cauter, PhD
Susan Sam, MD, MSc
M. Annette Miller, RN

**VA Puget Sound Health Care System and University of Washington (Seattle, WA)**

Steven E. Kahn, MB, ChB*
Karen M. Atkinson, BSN, RN+
Jerry P. Palmer, MD
Kristina M. Utzschneider, MD
Tsige Gebremedhin, BS
Abigail Kernan-Schloss, BA
Alexandra Kozedub, MSN, ARNP
Brenda K. Montgomery, MSN, RN, CDE
Emily J. Morse, BS

**Indiana University School of Medicine and Richard L. Roudebush VA Medical Center (Indianapolis, IN)**

Kieren J. Mather, MD*
Tammy Garrett, RN+
Tamara S. Hannon, MD
Amale Lteif, MD
Aniket Patel MD
Robin Chisholm, RN
Karen Moore, RN
Vivian Pirics, RN
Linda Pratt, RN

**University of Colorado Denver/Children's Hospital Colorado (Denver, CO)**

Kristen J. Nadeau, MD, MS*
Susan Gross, RD+
Philip S. Zeitler, MD, PhD
Jayne Williams, RN, MSN, CPNP
Melanie Cree-Green, MD, PhD
Yesenia Garcia Reyes, MS
Krista Vissat, RN, MSN, CPNP

**UPMC Children's Hospital of Pittsburgh (Pittsburgh, PA)**

Silva A. Arslanian, MD*
Kathleen Brown, RN, CDE+
Nancy Guerra, CRNP
Kristin Porter, RN, CDE

**Yale University (New Haven, CT)**

Sonia Caprio, MD*
Mary Savoye, RD, CDE+
Bridget Pierpont, MS+

**University of Southern California Keck School of Medicine/Kaiser Permanente Southern California (Los Angeles, CA)**

Thomas A. Buchanan, MD*
Anny H. Xiang, PhD*
Enrique Trigo, MD+
Elizabeth Beale, MD
Ting Chow, MPH
Fadi N. Hendee, MD
Namir Katkhouda, MD
Krishan Nayak, PhD
Mayra Martinez, MPH
Cortney Montgomery, BS
Xinhui Wang, PhD
Jun Wu, MS

**George Washington University Biostatistics Center (RISE Coordinating Center; Rockville, MD)**

Sharon L. Edelstein, ScM*
John M. Lachin, ScD
Ashley Hogan Tjaden, MPH
Mark T. Tripputi, PhD

**Northwest Lipid Research Laboratories (Central Biochemistry Laboratory; Seattle, WA)**

Santica Marcovina, PhD*
Jessica Harting+
John Albers, PhD

**Belmar Pharmacy (Drug Distribution Center; Lakewood, CO)**

Dave Hill

**NIH/NIDDK (Bethesda, MD**)

Peter J. Savage, MD
Ellen W. Leschek, MD
