# Supplementary material for: Islet Autoimmunity in Adults With Impaired Glucose Tolerance and Recently Diagnosed, Treatment Naïve Type 2 Diabetes in the Restoring Insulin SEcretion (RISE) Study
Source: Front Immunol. 2021 Apr 26;12:640251. doi: 10.3389/fimmu.2021.640251 (PMC8108986; doi:10.3389/fimmu.2021.640251)
Supplement: Supplementary file 1 [file Data_Sheet_1.pdf]

Supplemental Table 1. Baseline and follow-up characteristics of participants stratified by diabetes status at baseline and separated by baseline and month-12 GADA results.

Stratified by Baseline GADA Results N=213										
	Type 2 diabetes N=62					IGT N=151				
	Negative		Positive			Negative		Positive		
	N		N		p-value*	N		N		p-value*
**Demographics**										
**Age** (years)	61	54.8 ± 9.1	1	57.0	0.812	144	53.7 ± 8.7	7	54.7 ± 8.8	0.754
**Sex**					0.251					0.389
% Men		35 (57.4%)		0 (0.0%)			79 (54.9%)		5 (71.4%)	
% Women		26 (42.6%)		1 (100.0%)			65 (45.1%)		2 (28.6%)	
**Race**					0.478					0.061
% White		33 (54.1%)		0 (0.0%)			81 (56.3%)		1 (14.3%)	
% Black		17 (27.9%)		1 (100.0%)			39 (27.1%)		4 (57.1%)	
% Hispanic		8 (13.1%)		0 (0.0%)			13 (9.0%)		2 (28.6%)	
% Other		3 (4.9%)		0 (0.0%)			11 (7.6%)		0 (0.0%)	
**Baseline Anthropometrics**										
Weight (kg)	61	99.6 ± 18.7	1	88.5	0.557	144	103.3 ± 20.3	7	101.5 ± 10.5	0.821
Waist (cm)	59	109.4 ± 12.4	1	93.8	0.218	142	113.5 ± 14.3	7	106.6 ± 5.9	0.206
BMI (kg/m^2)	61	34.2 ± 5.7	1	31.1	0.582	144	35.6 ± 5.9	7	33.6 ± 2.9	0.384
**M12 Anthropometrics**										
Weight (kg)	61	96.2 ± 19.0	1	83.7	0.515	144	100.1 ± 21.1	5	101.3 ± 11.0	0.904
Waist (cm)	61	107.3 ± 13.2	1	93.1	0.290	142	111.1 ± 14.0	5	111.0 ± 6.9	0.990
BMI (kg/m2)	61	33.0 ± 5.7	1	29.4	0.530	144	34.4 ± 6.1	5	34.6 ± 3.0	0.963
**Stratified by GADA Status over 12 Months N=213**										
	Type 2 diabetes N=62					IGT N=151				
	Negative		Positive			Negative		Positive		
	N		N		p-value†	N		N		p-value†
**Demographics**										
**Age** (years)	61	54.8 ± 9.1	1	57.0	0.812	143	53.6 ± 8.7	8	55.3 ± 8.3	0.607
**Sex**					0.251					0.257
% Men		35 (57.4%)		0 (0.0%)			78 (54.5%)		6 (75.0%)	
% Women		26 (42.6%)		1 (100.0%)			65 (45.5%)		2 (25.0%)	
**Race**					0.478					0.034
% White		33 (54.1%)		0 (0.0%)			81 (56.6%)		1 (12.5%)	
% Black		17 (27.9%)		1 (100.0%)			38 (26.6%)		5 (62.5%)	
% Hispanic		8 (13.1%)		0 (0.0%)			13 (9.1%)		2 (25.0%)	
% Other		3 (4.9%)		0 (0.0%)			11 (7.7%)		0 (0.0%)	
**Baseline Anthropometrics**										
Weight (kg)	61	99.6 ± 18.7	1	88.5	0.557	143	103.2 ± 20.4	8	103.3 ± 11.0	0.987
Waist (cm)	59	109.4 ± 12.4	1	93.8	0.218	141	113.5 ± 14.3	8	106.9 ± 5.5	0.199
BMI (kg/m^2)	61	34.2 ± 5.7	1	31.1	0.582	143	35.6 ± 5.9	8	33.6 ± 2.7	0.347
**M12 Anthropometrics**										
Weight (kg)	61	96.2 ± 19.0	1	83.7	0.515	143	100.0 ± 21.1	6	103.8 ± 11.7	0.662
Waist (cm)	61	107.3 ± 13.2	1	93.1	0.290	141	111.1 ± 14.0	6	110.7 ± 6.2	0.940
BMI (kg/m2)	61	33.0 ± 5.7	1	29.4	0.530	143	34.5 ± 6.1	6	34.4 ± 2.7	0.992

Data are n (%) or mean ± SD. "Other" for race/ethnicity includes mixed race/ethnicity, Asian, American Indian, and other.

*** P values represent comparisons by baseline positivity within diabetes status stratum using ANOVA for continuous variables and Pearson Chi Square for categorical variables. All analyses are unadjusted.**

† P values represent comparisons by positivity over 12 months within diabetes status stratum using ANOVA for continuous variables and Pearson Chi Square for categorical variables. All analyses are unadjusted. Negative results are negative at baseline and month-12 samples. Positive responses are positive at either baseline, 12 months, or baseline and 12 months.

Supplemental Table 2. Baseline and follow-up characteristics of participants stratified by diabetes status at baseline and separated by baseline and month-12 T cell results.

Stratified by Baseline T Cell Results N=190										
	Type 2 diabetes N=56					IGT N=134				
	Negative (0-3)		Positive (4-18)			Negative (0-3)		Positive (4-18)		
	N		N		p-value*	N		N		p-value*
**Demographics**										
**Age** (years)	28	53.4 ± 9.7	28	55.8 ± 9.0	**0.336**	53	53.4 ± 8.5	81	54.5 ± 8.6	**0.463**
**Sex**					**0.786**					**0.235**
% Men	17	60.7%	16	57.1%		33	62.3%	42	51.9%	
% Women	11	39.3%	12	42.9%		20	37.7%	39	48.1%	
**Race**					**0.304**					**0.546**
% White	15	53.6%	12	42.9%		29	54.7%	43	53.1%	
% Black	7	25.0%	13	46.4%		17	32.1%	21	25.9%	
% Hispanic	5	17.9%	3	10.7%		5	9.4%	9	11.1%	
% Other	1	3.6%	0	0.0%		2	3.8%	8	9.9%	
**Baseline Anthropometrics**										
Weight (kg)	28	96.3 ± 16.1	28	104.4 ± 18.4	**0.084**	53	106.2 ± 18.9	81	98.5 ± 19.7	**0.027**
Waist (cm)	26	106.7 ± 10.7	28	112.6 ± 12.9	**0.074**	52	114.7 ± 12.2	80	109.3 ± 13.5	**0.020**
BMI (kg/m^2)	28	33.5 ± 5.8	28	35.3 ± 5.3	**0.236**	53	35.9 ± 5.0	81	34.1 ± 5.6	**0.074**
**M12 Anthropometrics**										
Weight (kg)	28	93.0 ± 15.7	25	101.4 ± 19.1	**0.086**	53	102.4 ± 20.2	64	97.8 ± 20.3	**0.219**
Waist (cm)	28	104.9 ± 11.6	25	111.8 ± 13.1	**0.048**	53	111.9 ± 12.5	62	109.8 ± 13.4	**0.408**
BMI (kg/m2)	28	32.4 ± 5.6	25	34.0 ± 5.3	**0.276**	53	34.6 ± 5.5	64	33.6 ± 5.7	**0.363**
**Stratified by T Cell Status over 12 Months N=203**										
	Type 2 diabetes N=57					IGT N=146				
	Negative		Positive			Negative		Positive		
	N		N		p-value†	N		N		p-value†
**Demographics**										
**Age** (years)	18	51.9 ± 8.6	39	55.8 ± 9.4	**0.146**	25	52.2 ± 9.7	121	54.1 ± 8.3	**0.308**
**Sex**					**0.738**					**0.023**
% Men	11	61.1%	22	56.4%		19	76.0%	62	51.2%	
% Women	7	38.9%	17	43.6%		6	24.0%	59	48.8%	
**Race**					**0.123**					**0.872**
% White	11	61.1%	16	41.0%		13	52.0%	65	53.7%	
% Black	3	16.7%	17	43.6%		8	32.0%	33	27.3%	
% Hispanic	4	22.2%	4	10.3%		3	12.0%	13	10.7%	
% Other	0	0.0%	2	5.1%		1	4.0%	10	8.3%	
**Baseline Anthropometrics**										
Weight (kg)	18	96.7 ± 15.3	39	101.9 ± 18.3	**0.302**	25	103.1 ± 16.2	121	102.5 ± 20.7	**0.884**
Waist (cm)	17	107.6 ± 10.4	38	110.6 ± 12.8	**0.394**	24	113.6 ± 11.9	120	112.2 ± 14.0	**0.637**
BMI (kg/m^2)	18	33.7 ± 6.0	39	34.8 ± 5.4	**0.513**	25	34.0 ± 4.5	121	35.6 ± 5.9	**0.223**
**M12 Anthropometrics**										
Weight (kg)	18	93.2 ± 15.7	36	98.4 ± 18.6	**0.311**	25	99.4 ± 18.4	104	101.4 ± 21.4	**0.662**
Waist (cm)	18	104.6 ± 11.0	36	109.7 ± 13.2	**0.168**	25	109.2 ± 11.8	102	112.4 ± 14.0	**0.296**
BMI (kg/m2)	18	32.5 ± 5.9	36	33.4 ± 5.2	**0.565**	25	32.8 ± 5.2	104	35.0 ± 6.1	**0.094**

Data are n (%) or mean ± SD. "Other" for race/ethnicity includes mixed race/ethnicity, Asian, American Indian, and other.

*** P values represent comparisons by baseline positivity within diabetes status stratum using ANOVA for continuous variables and Pearson Chi Square for categorical variables. All analyses are unadjusted.**

† **P values represent comparisons by positivity over 12 months within diabetes status stratum using ANOVA for continuous variables and Pearson Chi Square for categorical variables. All analyses are unadjusted. Negative results are negative at baseline and month-12 samples. Positive responses are positive at either baseline, 12 months, or baseline and 12 months.**

Supplemental Table 3. Relationship of GADA positivity with month-12 outcomes in participants with type 2 diabetes and participants with IGT.

	**Adjusted Mean**	**95% Confidence Limits**		
**Positivity***	**BMI (kg/m²)**	**Lower CI**	**Upper CI**	**p-value†**
IGT, Positive	35.01	33.62	36.39	0.2004
IGT, Negative	34.08	33.70	34.46	
T2D, Positive	31.56	27.81	35.31	0.5880
T2D, Negative	32.58	31.89	33.26	
	**HbA1c (%)**	**95% Confidence Limits**		**p-value**
IGT, Positive	5.74	5.55	5.93	0.1873
IGT, Negative	5.61	5.56	5.66	
T2D, Positive	5.59	4.68	6.50	0.9048
T2D, Negative	5.64	5.48	5.81	
	**Fasting Glucose (mg/dL)**	**95% Confidence Limits**		**p-value**
IGT, Positive	107.82	100.55	115.10	0.4261
IGT, Negative	104.81	102.81	106.81	
T2D, Positive	102.26	77.75	126.77	0.7658
T2D, Negative	105.91	101.42	110.40	
	**Fasting C-Peptide (ng/mL)**	**95% Confidence Limits**		**p-value**
IGT, Positive	3.62	2.74	4.51	0.4293
IGT, Negative	3.26	3.01	3.51	
T2D, Positive	2.52	-0.05	5.10	0.6476
T2D, Negative	3.11	2.64	3.58	
	**Fasting Insulin (uU, mL)**	**95% Confidence Limits**		**p-value**
IGT, Positive	17.02	10.76	23.29	0.2919
IGT, Negative	13.59	11.85	15.32	
T2D, Positive	10.92	-10.61	32.45	0.9523
T2D, Negative	11.56	7.63	15.50	
	**2-Hr Glucose (mg/dL)**	**95% Confidence Limits**		**p-value**
IGT, Positive	172.49	143.12	201.86	0.6595
IGT, Negative	165.77	157.62	173.92	
T2D, Positive	212.12	124.48	299.76	0.7810
T2D, Negative	199.96	183.90	216.02	
	**M/I (mg/kg/min/pg/L)**	**95% Confidence Limits**		**p-value**
IGT, Positive	3.04	1.79	5.14	0.6586
IGT, Negative	3.42	3.00	3.91	
T2D, Positive	5.84	1.26	27.16	0.4291
T2D, Negative	3.17	2.40	4.19	
	**Steady State C-Peptide (ng/mL)**	**95% Confidence Limits**		**p-value**
IGT, Positive	4.39	3.62	5.33	0.6876
IGT, Negative	4.57	4.35	4.80	
T2D, Positive	4.16	2.41	7.17	0.9510

T2D, Negative	4.23	3.83	4.66	
	**ACPRg (ng/mL)**	**95% Confidence Limits**		**p-value**
IGT, Positive	2.25	1.85	2.73	0.6628
IGT, Negative	2.15	2.05	2.26	
T2D, Positive	1.71	1.05	2.77	0.7723
T2D, Negative	1.83	1.68	2.00	
	**ACPRmax (ng/mL)**	**95% Confidence Limits**		**p-value**
IGT, Positive	4.62	3.37	6.34	0.9707
IGT, Negative	4.60	4.24	4.99	
T2D, Positive	4.22	2.45	7.26	0.6416
T2D, Negative	3.71	3.37	4.10	
	**Insulinogenic Index**	**95% Confidence Limits**		**p-value**
IGT, Positive	163.04	110.21	215.87	0.1596
IGT, Negative	124.19	109.29	139.09	
T2D, Positive	17.28	-175.72	210.28	0.4484
T2D, Negative	90.66	55.16	126.15	
	**C-Peptide Index**	**95% Confidence Limits**		**p-value**
IGT, Positive	9.89	7.06	12.72	0.5835
IGT, Negative	9.09	8.30	9.88	
T2D, Positive	4.00	-8.00	16.00	0.5300
T2D, Negative	7.77	5.56	9.97	

Adjusted means for values at month 12 visits. All models are adjusted for age, sex, race/ethnicity, treatment group, and baseline value of dependent variable. All models except for BMI are also adjusted for baseline BMI. Models for SSCP, ACRPg, and ACPRmax are also adjusted for baseline and month-12 insulin sensitivity (M/I). Analyses for M/I, SSCP, ACPRg, and ACPRmax performed using the log value.

***Positivity over 12 months: Negative results are negative at baseline and month-12 samples. Positive responses are positive at either baseline, 12 months, or baseline and month-12.**

†P values indicate differences between autoimmune negative and positive participants within diabetes or IGT.
